# Patient-derived mutations within the N-terminal domains of p85α impact PTEN or Rab5 binding and regulation

**DOI:** 10.1038/s41598-018-25487-5

**Published:** 2018-05-08

**Authors:** Paul Mellor, Jeremy D. S. Marshall, Xuan Ruan, Dielle E. Whitecross, Rebecca L. Ross, Margaret A. Knowles, Stanley A. Moore, Deborah H. Anderson

**Affiliations:** 1 Cancer Research Group, University of Saskatchewan, 107 Wiggins Road, Saskatoon, Saskatchewan S7N 5E5 Canada; 2 Department of Biochemistry, University of Saskatchewan, 107 Wiggins Road, Saskatoon, Saskatchewan S7N 5E5 Canada; 3 Section of Experimental Oncology, Leeds Institute of Cancer and Pathology, St James’s University Hospital, Leeds, United Kingdom; 4 Cancer Research, Saskatchewan Cancer Agency, 107 Wiggins Road, Saskatoon, Saskatchewan S7N 5E5 Canada

## Abstract

The p85α protein regulates flux through the PI3K/PTEN signaling pathway, and also controls receptor trafficking via regulation of Rab-family GTPases. In this report, we determined the impact of several cancer patient-derived p85α mutations located within the N-terminal domains of p85α previously shown to bind PTEN and Rab5, and regulate their respective functions. One p85α mutation, L30F, significantly reduced the steady state binding to PTEN, yet enhanced the stimulation of PTEN lipid phosphatase activity. Three other p85α mutations (E137K, K288Q, E297K) also altered the regulation of PTEN catalytic activity. In contrast, many p85α mutations reduced the binding to Rab5 (L30F, I69L, I82F, I177N, E217K), and several impacted the GAP activity of p85α towards Rab5 (E137K, I177N, E217K, E297K). We determined the crystal structure of several of these p85α BH domain mutants (E137K, E217K, R262T E297K) for bovine p85α BH and found that the mutations did not alter the overall domain structure. Thus, several p85α mutations found in human cancers may deregulate PTEN and/or Rab5 regulated pathways to contribute to oncogenesis. We also engineered several experimental mutations within the p85α BH domain and identified L191 and V263 as important for both binding and regulation of Rab5 activity.

## Introduction

Phosphatidylinositol 3-kinase (PI3K) is an important signaling enzyme, acting downstream from many activated receptors, including receptor tyrosine kinases^1^. PI3K consists of a regulatory p85α protein and a catalytic p110 protein; their constitutive binding serves to stabilize the p110 protein and inhibit p110-PI3K activity^2^. The binding of p85α to newly formed phosphotyrosine sites on activated receptors, either directly or via adapter proteins, relieves the default inhibition of PI3K and also relocalizes p110-PI3K to the plasma membrane^3,4^. p110-PI3K phosphorylates phosphatidylinositol (PI) lipids such as PI4,5P_2_ converting it to PI3,4,5P_3_, an important lipid second messenger that recruits PH (pleckstrin homology) domain-containing proteins such as PDK1 (3-phosphoinositide-dependent protein kinase 1) and Akt to the plasma membrane^5^. Phosphorylation by PDK1 and mTORC2 activates Akt to phosphorylate numerous downstream target proteins that result in cell cycle progression, cell growth, migration and survival^6^.

PTEN (phosphatase and tensin homologue deleted on chromosome 10) counteracts PI3K signaling by dephosphorylating PI3,4,5P_3_ back to PI4,5P_2_. PTEN is differentially ubiquitinated, with monoubiquitination causing nuclear translocation, and polyubiquitination causing proteosomal degradation^7,8^. Nuclear PTEN has roles in chromosomal stability, DNA repair responses and cell cycle regulation^9^. Recent work has shown that in the absence of p110, the p85α protein homodimerizes^10^ and binds PTEN in response to growth factor stimulation to positively regulate PTEN lipid phosphatase activity^11^. Association with p85α has also been shown to reduce PTEN ubiquitination, thereby protecting PTEN from degradation and promoting PTEN protein stability^12^. Thus, p85α is a dual regulator of both the p110-PI3K and the PTEN-PI3 phosphatase controlling flux through the PI3K/PTEN pathway^13,14^.

**Figure 1:**
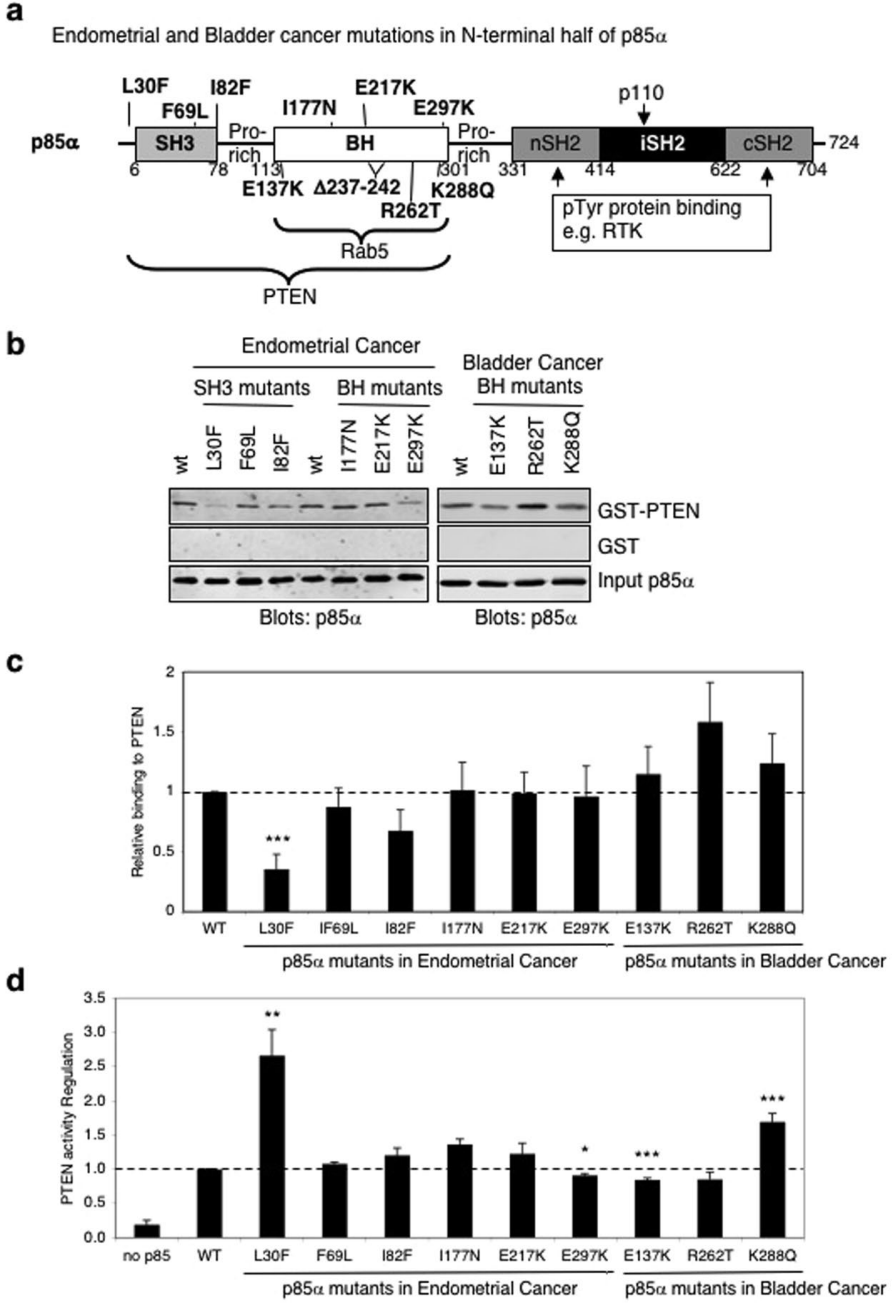
Binding and regulation of PTEN by p85α patient-derived SH3 and BH domain mutants. (**a**) Schematic representation of p85α with the locations of endometrial and bladder cancer-associated mutations within the SH3 and BH domains shown. (**b**) Pull-down assays for p85α mutants binding to PTEN. Input of p85α is 4% of the amount used in the pull-down binding experiments. Data representative of at least 9 independent experiments. Full-length images of the cropped blots are in Supplementary Fig. S4. (**c**) Quantification of binding assay results from panel b. Mean ± SEM. ***P < 0.001 as compared to wild type p85α. (**d**) The ability of each p85α mutant protein to regulate PTEN was determined using a PTEN lipid phosphatase activity assay. Mean ± SEM for 4–5 independent experiments. ***P < 0.001, **P < 0.01, *P < 0.05 as compared to wild type p85α.

The p85α protein has five domains that mediate interactions with different proteins (Fig. 1a). The N-terminal domains of p85α include an SH3 domain, capable of binding to proline-rich sequences^15^, and a BH (breakpoint cluster region homology) domain. The BH domain can bind to and regulate several GTPases including Rab5, Rac1 and Cdc42^16–18^. In addition, the p85α BH domain, either alone or together with the SH3 domain, can bind to and positively regulate PTEN activity^11^. The nSH2 and cSH2 domains of p85α bind to phosphotyrosine sites on upstream signaling proteins such as activated receptor tyrosine kinases (RTK)^19^. Between the SH2 domains is the p110 binding domain, also known as the inter-SH2 or iSH2 domain, and together with the nSH2 it binds p110 proteins^20^.

The critical balance of PI3K/PTEN signaling controls the magnitude and duration of downstream Akt signaling and is frequently disrupted in many types of cancer^1^. This occurs through gain-of-function mutations that cause activation of p110α, or through loss-of-function mutations or reduced levels of PTEN^21^. In addition, there are several cancer-associated p85α mutations in the C-terminal half of p85α that result in activated p110α^4,20,22–24^. These p85α mutations typically relieve the inhibitory effects yet retain the stabilizing interactions with p110α.

Both the N-terminal SH3 and BH domains of p85α contribute to PTEN binding^11^ and recent reports have now identified mutations within these regions in endometrial^12^ and bladder^25^ cancers. One endometrial cancer-associated mutation (I177N) was found to disrupt p85α homodimerization, resulting in reduced PTEN binding^10^. A second mutation (I133N) did not affect p85α homodimerization yet still showed reduced PTEN binding. Both mutations increased PTEN ubiquitination, and promoted downstream Akt phosphorylation^10^. Taken together, these results demonstrate that p85α directly binds to and blocks ubiquitination of PTEN to promote PTEN stability, and p85α also positively regulates the lipid phosphatase activity of PTEN. Mutations in p85α that reduce either of these effects could contribute to enhanced PI3K pathway activation and promote oncogenesis.

The BH domain of p85α has GAP (GTPase activating protein) activity towards Rab GTPases Rab5 and Rab4, with key roles in vesicle tethering, which is important for receptor trafficking and down-regulation processes^17,26,27^. The BH/GAP domain of p85α is similar in sequence^28^ and structure^29,30^ to other GAPs, and mutation of the highly conserved arginine finger residue, R151, compromises its Rab5 GAP activity^17^. Overexpression of p85α in cells also results in decreased Rab5-GTP levels, consistent with its role as a Rab5 GAP *in vivo*^31^.

Free p85α can decrease the amount of Rab5-GTP in a dose-dependent manner^32^, supporting its function as a Rab5 GAP. In addition, the p85α/p110β complex can compete with p85α for binding to Rab5-GTP, and this has been shown to block the Rab5 GAP activity of p85α^32^. A single point mutation within the BH domain (R274A) renders p85α Rab-GAP-defective and is oncogenic^33^ due to rapid trafficking of activated phosphorylated receptor complexes with reduced opportunities for sorting and lysosomal-mediated degradation^34^. Thus, mutations within the BH domain of p85α can be transforming through deregulation of Rab GTPase-mediated receptor trafficking.

In this report, we set out to determine if several p85α SH3 domain and BH domain mutations identified in human bladder and endometrial cancer tumors influence the binding and/or regulation of PTEN and Rab5.

## Results

### Impact of endometrial and bladder cancer-derived p85α BH domain mutations on PTEN binding and lipid phosphatase activity

Recent analyses of PI3K pathway mutations in human endometrial^12^ and bladder cancer samples^25^ identified a novel class of previously uncharacterized mutations within the N-terminal SH3 and BH domains of p85α (Fig. 1a). The mutations found in endometrial cancer included three within the SH3 domain (L30F, F69L and I82F) and three within the BH domain (I177N, E217K, E297K). The mutations found in bladder cancer included four within the BH domain (E137K, R262T, K288Q, in-frame deletion of 237–242). Since our previous studies showed that the p85α SH3 and BH domains are important for PTEN binding, and that p85α can upregulate PTEN lipid phosphatase activity^11^, these tumor-derived N-terminal p85α mutations may alter the binding and/or regulation of PTEN by p85α.

Each of the cancer-associated p85α point mutants was purified as a heterologously expressed protein from *E. coli*, except p85α-Δ237–242 that was unstable. Each mutant p85α protein was tested for its ability to bind to GST-PTEN in a pull-down binding assay (Fig. 1b,c), as well as to stimulate PTEN lipid phosphatase activity (Fig. 1d). The p85α-L30F mutant showed reduced steady state binding to PTEN yet had a stronger stimulatory effect on PTEN lipid phosphatase activity, both of which were significantly different from the p85α wild type protein. In contrast, several of the p85α mutants (E137K, K288Q, E297K) showed near wild type binding to PTEN (Fig. 1b), and either had enhanced (K288Q) or reduced (E297K, E137K) ability to stimulate PTEN lipid phosphatase activity (Fig. 1c,d). These results suggest that stable binding between mutant p85α proteins and PTEN do not consistently correlate with regulatory effects on PTEN activity. In addition, some of these tumor-derived p85α mutations altered the binding and/or regulation of PTEN.

To assess if these cancer-associated mutations altered the overall folding of p85α, circular dichroism (CD) spectroscopy was used to evaluate possible changes in protein secondary structure (Supplementary Fig. S1). The far UV CD spectrum for each of the mutants was similar to that for the wild type p85α protein, suggesting that the point mutations did not severely disrupt protein folding.

### Impact of endometrial and bladder cancer-derived p85α BH domain mutations on Rab5 binding and GTPase activity protein (GAP) activity

**Figure 2:**
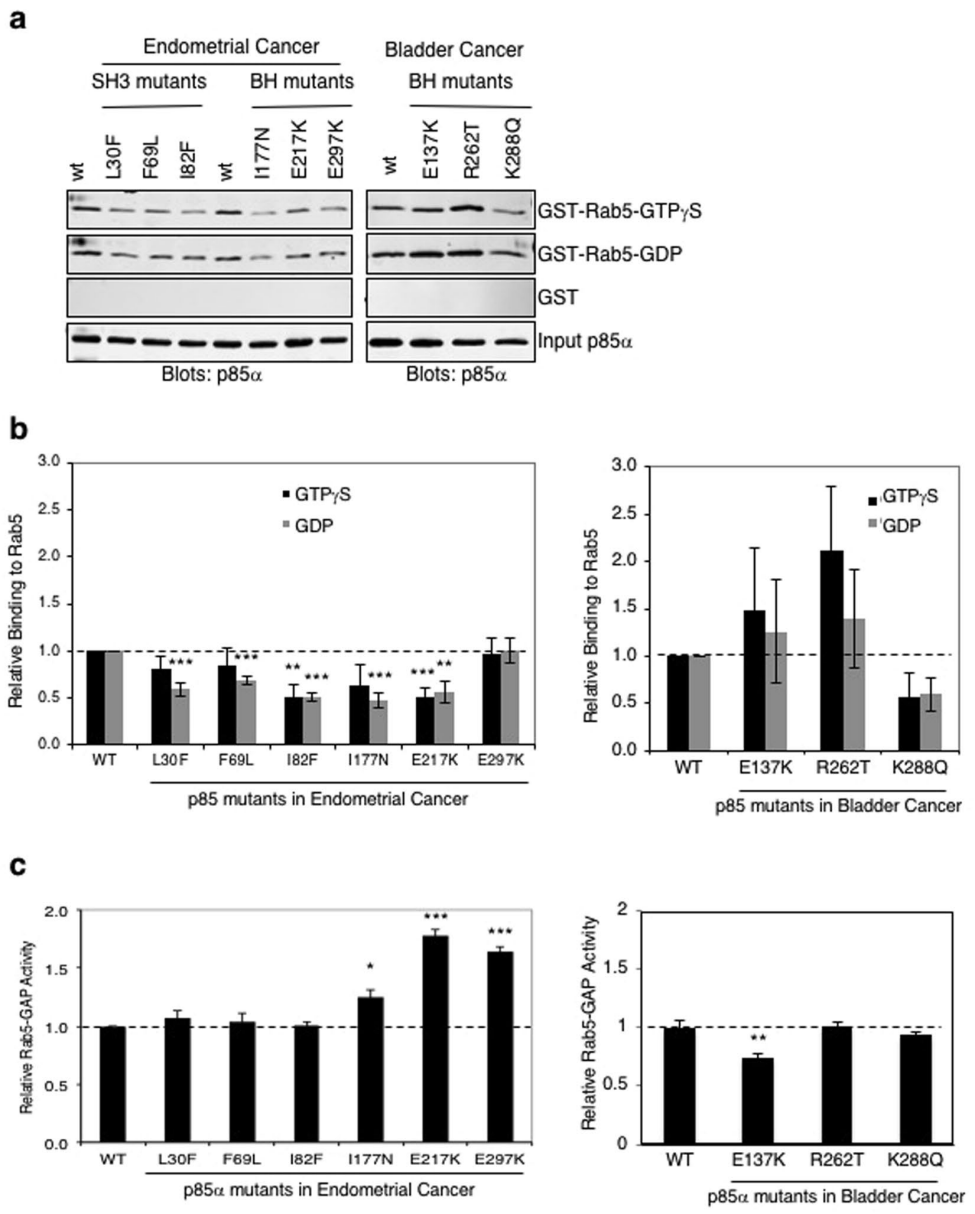
Binding and regulation of Rab5 by p85α patient-derived SH3 and BH domain mutants. (**a**) Pull-down assay with GST and GST-Rab5 mutants immobilized on glutathione Sepharose beads and loaded with the indicated nucleotide. GTPγS is a non-hydrolyzable analogue of GTP. Binding of purified p85α wild type or mutant protein was detected using an immunoblot analysis. Input of p85α is 4% of the amount used in the pull-down binding experiments. Data representative of at least 4 independent experiments. Full-length images of the cropped blots are in Supplementary Fig. S5. (**b**) Quantification of binding assay results from panel a. Mean ± SEM. ***P < 0.001, **P < 0.01 as compared to wild type p85α. (**c**) Rab5 was loaded with [α-^32^P]-GTP and analyzed for its GTPase activity either alone (no p85α) or in the presence of the indicated p85α protein to measure Rab5 GAP activity. Mean ± SEM for 3 independent experiments. ***P < 0.001, **P < 0.01, *P < 0.05 as compared to wild type p85α.

The ability of each p85α mutant to bind Rab5 was determined with a pull-down assay using GST-Rab5 immobilized on glutathione Sepharose beads and loaded with either a non-hydrolyzable GTP analogue (GTPγS), or GDP (Fig. 2a,b). Most of the endometrial cancer-associated p85α mutant proteins, as well as the K288Q bladder cancer-derived mutant, showed reduced binding to Rab5. In contrast, the bladder cancer-associated p85α mutants, E137K and R262T, showed a slightly enhanced Rab5 binding. The p85α mutants were also analyzed for their ability to regulate Rab5 GTPase activity, in a Rab5 GAP assay (Fig. 2c). Three BH domain mutants, I177N, E217K and E297K, all showed significantly increased Rab5-GAP activity, whereas the E137K mutant had reduced activity. Again, stable binding between mutant p85α proteins and Rab5 do not strictly correlate with the ability of p85α to regulate Rab5-GTPase activity.

### Structure of the bovine p85α BH domain and impact of several patient-derived mutations

**Figure 3:**
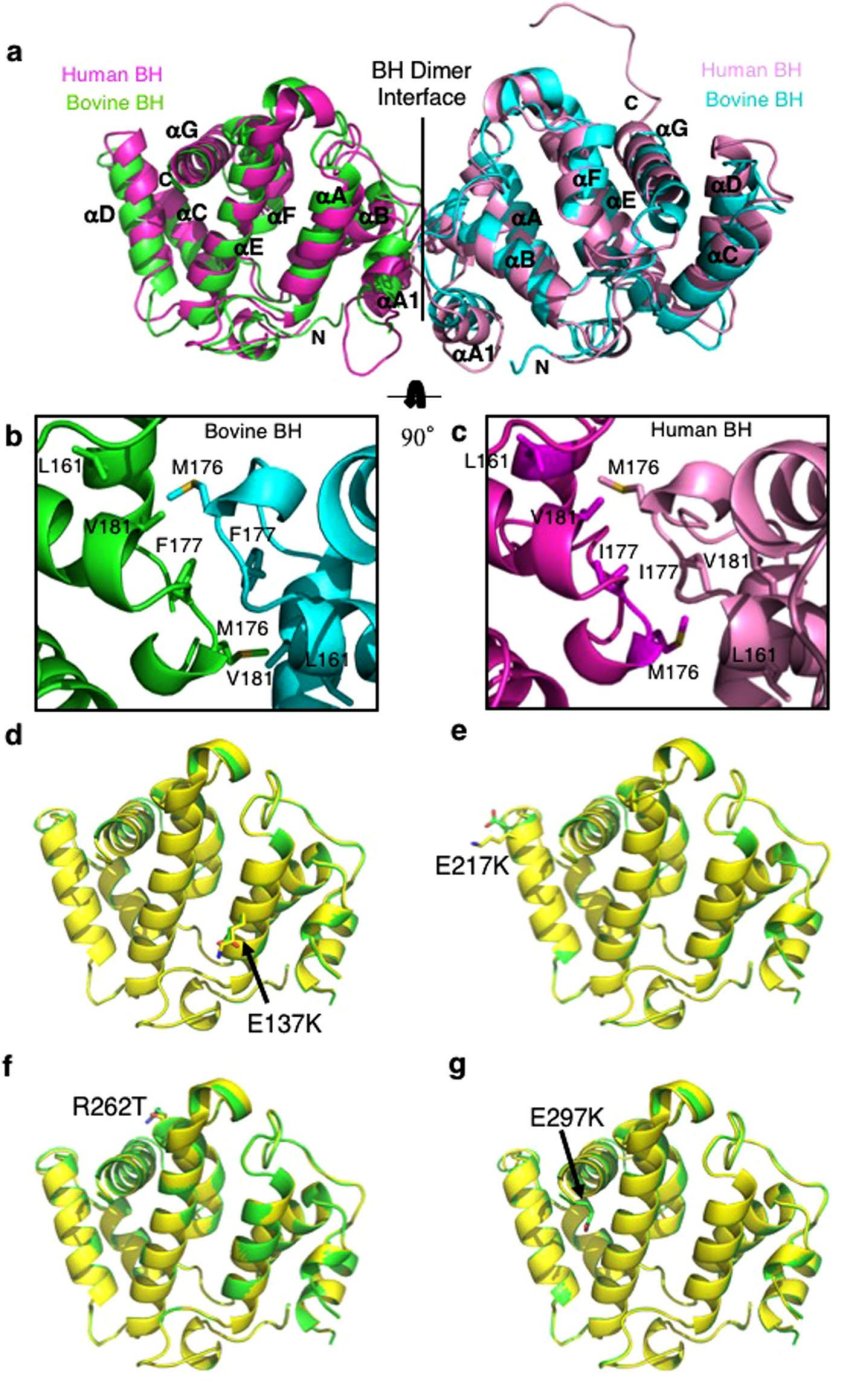
Crystal structures for bovine p85α (105–319) wild type and containing patient-derived mutations. (**a**) Overlay of the bovine p85α (105–319) crystal (green and cyan, resolution 2.25 Å) with the crystal structure for the human p85α (105–319) protein fragment (1PBW; magenta and pink, resolution 2.0 Å). A homodimer of the bovine p85α BH domains was visible containing residues 113–297 for both components of the dimer. (**b,c**) p85α BH domain residues involved in the hydrophobic dimerization interface within the crystal lattice of the bovine protein (**b**; L161, M176, F177 and V181) and the human protein (**c**; L161, M176, I177 and V181). (**d**–**g**) Overlays of the crystal structures for p85α (105–319); wild type (green) and cancer-associated point mutants (yellow): E137K mutant (**d**), E217K mutant (**e**), R262T mutant (**f**), E297K mutant (**g**). Wild type and mutant sidechains are shown in stick representation. Lack of density for the K297 sidechain prevented inclusion of the sidechain, and it is modeled as Ala in the structure (**e**).

We crystalized the wild type bovine p85α BH domain (105–319) and solved its structure to 2.25 Å resolution. X-ray diffraction data collection and structure refinement statistics for bovine p85α (105–319) wild type is provided in Supplementary Table S1. The bovine p85α BH domain has a very similar structure to the human p85α BH domain (105–319) structure previously solved^29^ (Fig. 3a). These structures have a root-mean-square difference of 1.22 Å for backbone atoms and a 93% (199 of 215 amino acids) sequence identity. The BH domain dimers observed within the crystal structures are mediated by reciprocal interactions between M176 from one monomer fitting into a hydrophobic pocket created by L161, 177 (Ile in human, Phe in bovine), and V181 in the other monomer (Fig. 3b,c). The buried surface area in the bovine p85α BH–BH crystal lattice is 535 Å^2^, similar to in the human p85α BH–BH crystal lattice (527 Å^2^)^10^. To determine if the patient-derived p85α BH domain mutations impacted the overall folded structure of the BH domain, several of these BH domain mutants were crystalized and their structures were determined using X-ray crystallography (Fig. 3d–g). X-ray diffraction data collection and structure refinement statistics for bovine p85α (105–319) mutants (E137K, E217K, R262T, E297K) are provided in Supplementary Table S1. The structures of the BH domains were not perturbed by any of the patient-derived mutations tested (E137K, E217K, R262T, E297K), suggesting that the impact of these mutations on PTEN and/or Rab5 binding and regulation was not due to aberrant BH domain folding.

### Defining key p85α BH domain residues required for Rab5 binding

**Figure 4:**
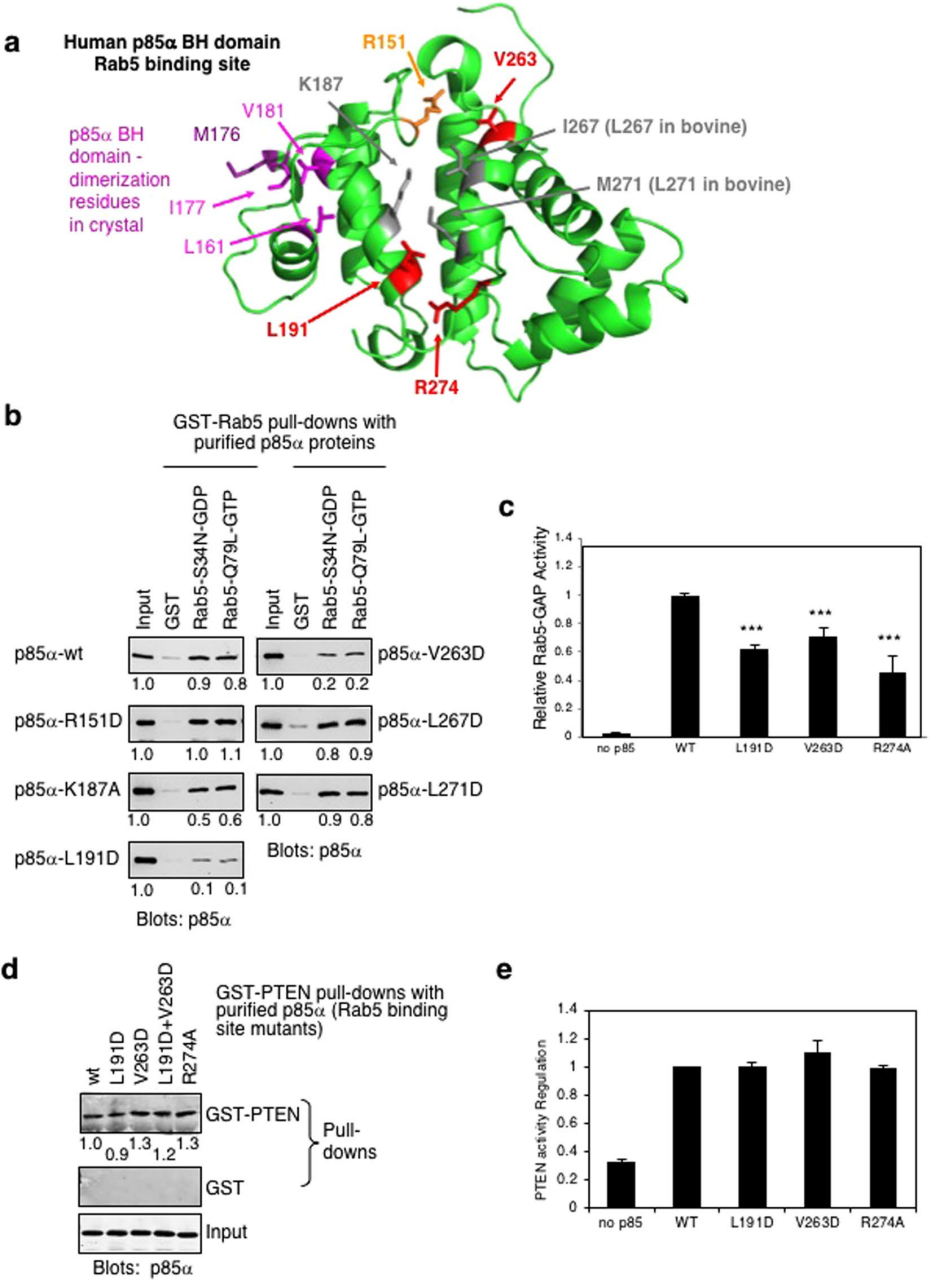
Residues L191 and V263 in the p85α BH domain are important for Rab5 binding. (**a**) The BH domain of human p85α with the proposed G protein binding (i.e. Rab5) residues indicated. Residues with little or no effect on Rab5 binding are shown in grey (K187, I267 [L267 in bovine p85α], M271 [L271 in bovine p85α]), with catalytically important R151 in orange. Residues important for both Rab5 binding and catalytic activity are shown in red (L191, V263, R274). Residues that help mediate BH–BH domain dimerization within the crystal structure are shown in M176 (purple) from one BH domain fitting into a hydrophobic pocket containing L161, I177 and V181 (pink) on the other BH domain^29^. (**b**) Pull-down assay with GST and GST-Rab5 mutants immobilized on glutathione Sepharose beads and loaded with the indicated nucleotide. Binding of purified p85α wild type or mutant protein was detected using an immunoblot analysis. The input lanes contain 0.4% of the purified p85α protein used in the pull-down assay. Full-length images of the cropped blots are in Supplementary Fig. S6. (**c**) The ability of each p85α mutant protein to regulate Rab5 GTPase activity was determined using a Rab5 GAP assay. Mean ± SEM from three independent experiments. ***P < 0.001 as compared to wild type p85α. (**d**) Immobilized GST and wild type GST-PTEN were allowed to bind purified p85α wild type (wt) or mutant proteins as indicated. The input lanes contain 0.4% of the purified p85α protein used in the pull-down assay. Full-length images of the cropped blots are in Supplementary Fig. S6. (**e**) PTEN lipid phosphatase activity was measured either alone (no p85α) or with the added p85α WT or mutant protein. Mean ± SEM from five independent assays. No significant differences were measured for the mutants as compared to wild type p85α.

The crystal structure of the human p85α BH domain has previously been reported and several residues were proposed to form the G protein binding site (Fig. 4a)^29^. These residues included several that were highly conserved within the BH/GAP domain family (R151, K187, P270 and R274; Supplementary Fig. S2). Two hydrophobic residues (I267 and M271; both are Leu in the bovine sequence) were also suggested to contribute to the hydrophobic characteristics of this proposed binding site, as well as two residues that were highly conserved between other GAP domains but divergent in p85α BH domains across multiple species (L191, V263; Supplementary Fig. S2)^29^. To define the amino acids involved in Rab5 binding, engineered mutations within the context of bovine p85α were generated and assessed in a pull-down binding assay. The assay used two Rab5 mutants, one that preferentially bound GDP (S34N), and the other that lacked GTPase activity and was thus locked in a GTP-bound conformation (Q79L)^35^. Several of the p85α BH domain mutations had little or no effect on Rab5 binding, including K187A, L267D (bovine residue corresponding to human I267), L271D (bovine residue corresponding to human M271) (Fig. 4b). The p85α-R151D mutant also retained Rab5 binding, similar to the p85α-R151A mutant tested previously^17^. Two p85α mutations (L191D and V263D) caused a large reduction in binding to Rab5 (Fig. 4b). In each case the observed binding was similar for both Rab5-S34N-GDP and Rab5-Q79L-GTP, suggesting these p85α mutations did not distinguish between the two conformations of Rab5. Consistent with their reduced binding ability, p85α-L191D and p85α-V263D also showed reduced Rab5 GAP activity (Fig. 4c). These results showed that in addition to the R274 residue^17^, both L191 and V263 are important residues within the BH domain of p85α for Rab5 binding and regulation, as well as R151 that is important for p85α GAP activity.

We have also shown that the BH domain of p85α can bind directly to PTEN, and together with the SH3 domain of p85α, is important for PTEN binding^11^. To determine if p85α BH domain mutations that impact Rab5 binding and regulation (L191D, V263D, R274A) also influence PTEN binding, these p85α mutants were tested for their ability to bind to immobilized GST-PTEN in a pull-down assay; a double mutant p85α-L191D + V263D was also generated and tested (Fig. 4d). These mutations had little or no effect on PTEN binding, or on the ability of p85α to enhance PTEN lipid phosphatase activity (Fig. 4e). The data suggest that PTEN and Rab5 bind to different surfaces of the BH domain of p85α.

## Discussion

The *PIK3R1* gene encodes p85α and two smaller isoforms p55α and p50α (both lacking the N-terminal SH3 and BH domains)^36^. *PIK3R1* mutations have been shown to occur at high frequency (25%) in endometrial cancer^12,37^ and at lower frequencies in several other cancers, including glioblastomas (7–10%^22,38^), colorectal cancers (8%^23^) and bladder cancer (6%^25^). Additional efforts have been made to determine the frequency and role of *PIK3R1* mutations in cancer^10,25,38–40^. The most common p85α mutation sites include those within the nSH2 domain (R348, G376, L380) and iSH2 domain (K459, D560, N564 and R574)^23^. They are gain-of-function mutations resulting in a loss of p110α inhibition and enhanced PI3K signaling^23,41–43^.

**Figure 5:**
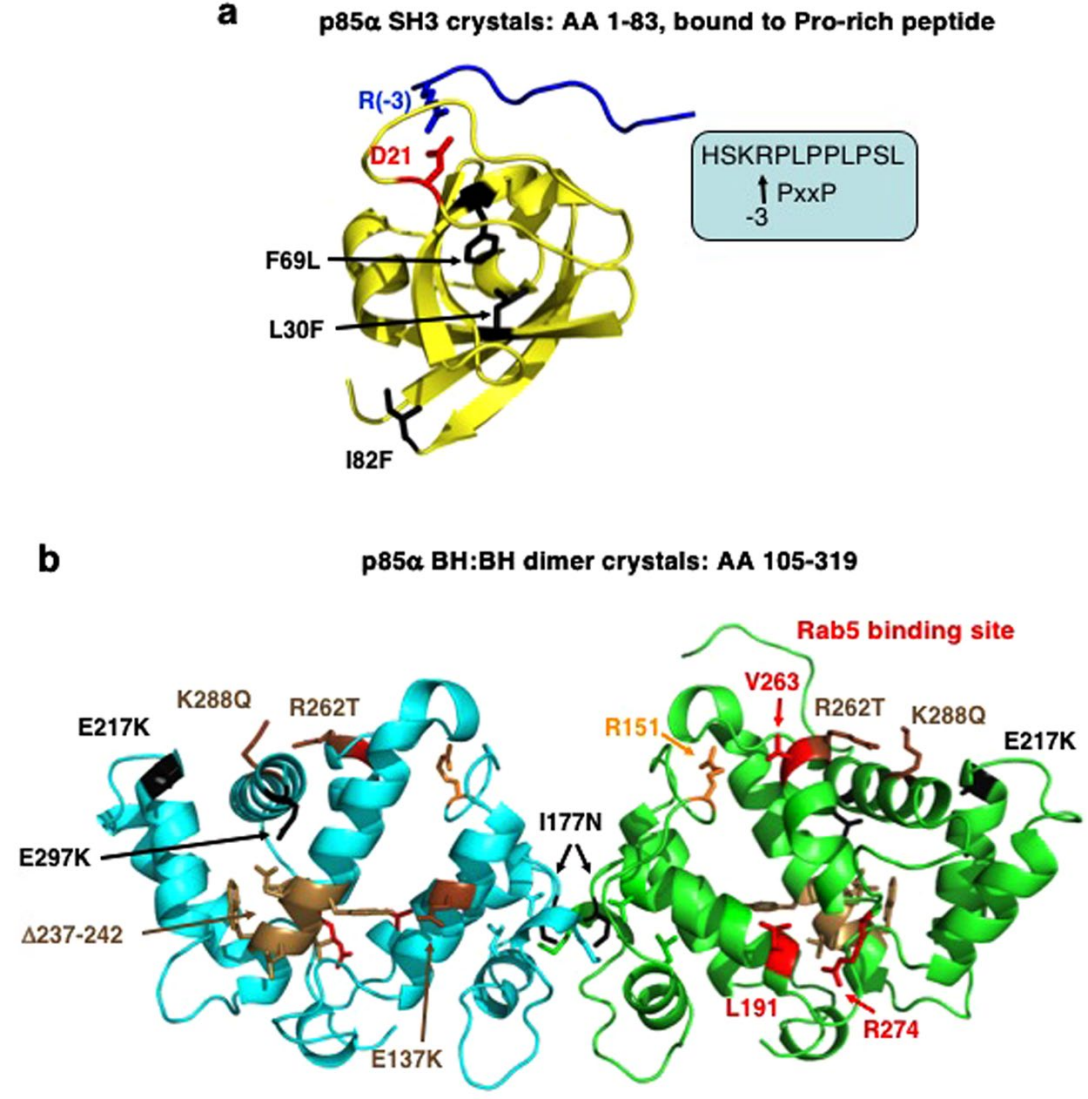
Structures and locations of key residues within the SH3 and BH domains of p85α. (**a**) The human p85α SH3 domain with endometrial cancer-associated mutations shown (black) in relation to D21 (red) important for binding to proline-rich peptides containing a key arginine residue (blue)^53^. (**b**) The bovine p85α BH domain showing the residues that are important for Rab5 binding (red) and Rab5-GAP activity (red, orange). Endometrial cancer patient-derived mutations (black) and bladder cancer-associated mutations (brown) are also shown, including an in-frame deletion (Δ237–242), which was too unstable to purify. I177 (black) is both involved in BH–BH domain dimerization within the crystal structure and is a residue mutated in endometrial cancer.

This study focused on characterizing several endometrial and bladder cancer patient-derived mutations located within the N-terminal half of p85α containing the SH3 and BH domains. The location of these mutations within each of these domain structures is shown in Fig. 5. Several patient-derived mutations within the BH domain of p85α can enhance PTEN binding (e.g. R262T) that may help to stabilize PTEN by blocking its ubiquitination^10^. Other BH domain mutants stimulate PTEN activity either less (E137K) or more (K288Q) than wild type p85α, suggesting they could alter PI3K/PTEN pathway regulation. Consistent with the marked increase in the stimulation of PTEN activity for p85α-K288Q, cells expressing this mutant show reduced pAkt levels^25^.

Our analysis using purified p85α and PTEN proteins showed that the p85α-I177N mutant retained its ability to both bind and regulate PTEN, similar to that of wild type p85α. This is in contrast to the proposed role for the I177 residue within the BH–BH dimer interface noted in the crystallized protein^29^, but was in good agreement with a recent study suggesting that in solution BH domains do not typically interact^44^. Cheung *et al*. found decreased p85α-I177N dimer formation with wild type p85α, and decreased PTEN binding with a corresponding ~2–3 fold increase in pAkt in cells as compared to cells expressing wild type p85α^10^. It is possible that other protein partners or cellular factors may influence p85α dimerization and PTEN interactions in the context of these cell-based analyses that are not present when analyzing purified proteins.

Monomeric p85α has been shown to bind, regulate and stabilize p110α^2–4^, whereas dimeric p85α is required to bind, regulate and stabilize PTEN^10^. Given the importance of SH3 domain–proline-rich region interaction in mediating p85α dimerization, cancer patient-derived mutations within the SH3 domain might disrupt dimerization and thus the ability of the mutant protein to bind and/or regulate PTEN. Interestingly, the p85α-L30F mutant protein showed reduced steady state binding to PTEN, but this resulted in enhanced ability to stimulate PTEN lipid phosphatase activity. These contrasting results suggest that reductions in stable interactions between a p85α mutant and PTEN may increase the influence of the p85α mutant perhaps by allowing it to interact with more PTEN molecules in a given amount of time.

**Figure 6:**
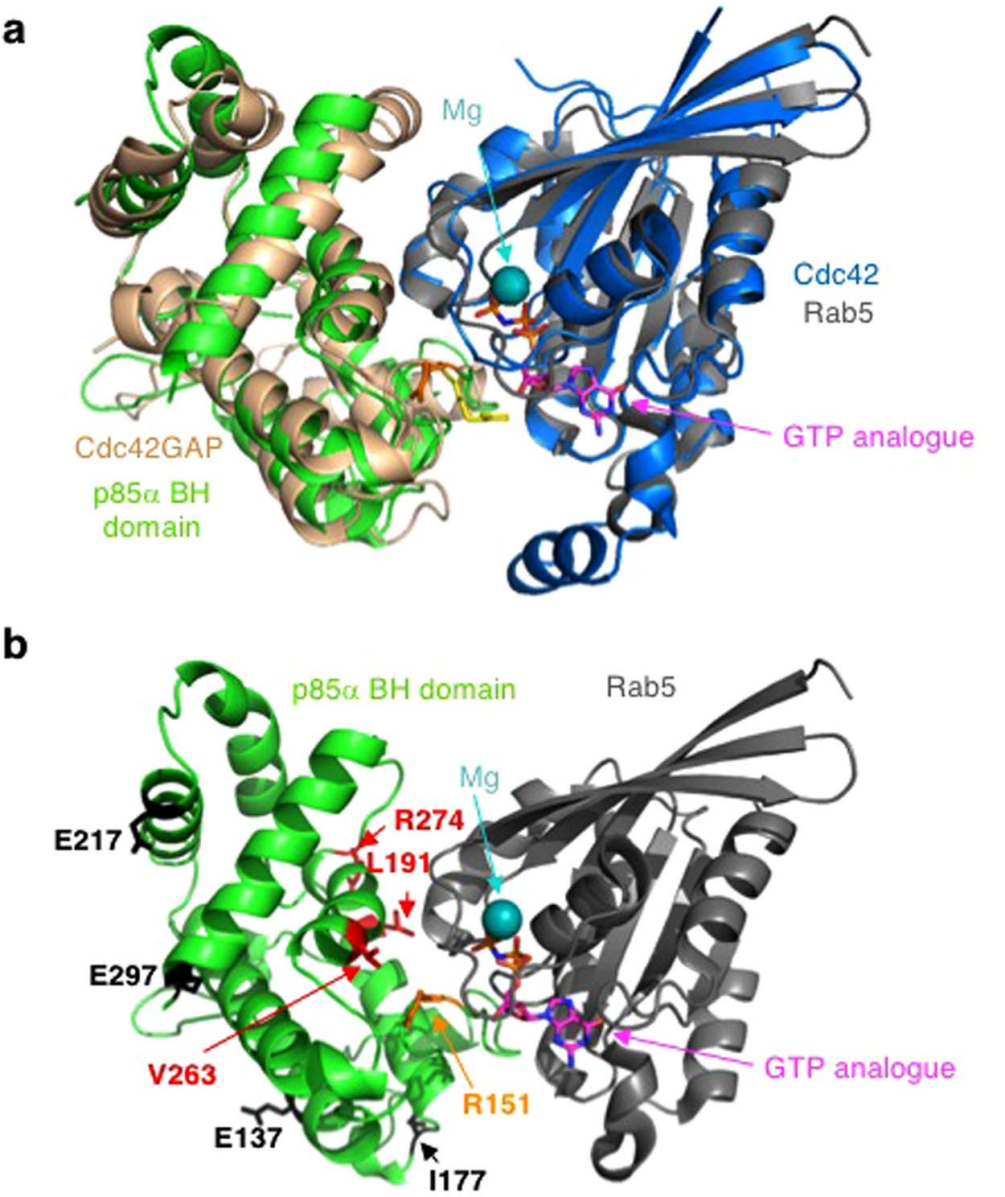
Modeled interface region between the human p85α BH domain and Rab5. (**a**) Overlay of the crystal structure of human Cdc42GAP (tan) – Cdc42 (blue) complex (PDB ID: 2NGR) with the human p85α BH domain (green; PDB ID: 1PBW) and the human Rab5-GTP analogue (15–184; grey; PDB ID: 1R2Q). The magnesium (teal) and GTP analogue (magenta) bound to Rab5 are indicated. Catalytically important arginine residues within Cdc42GAP (yellow) and p85α BH domain (orange) are shown. (**b**) Modeled human p85α BH domain (green) – Rab5 (grey) complex. The magnesium (teal) and GTP analogue (magenta) bound to Rab5 are indicated. Key p85α BH domain residues are shown: R151 (orange, important for GAP activity), and residues in red are important for Rab5 binding (L191, V263, R274). The location of patient-derived p85α BH mutations with significant impacts on Rab5 binding are shown in black (E137, I177, E217, E297).

The p85α BH domain also exhibits Rab5 GAP activity to stimulate Rab5-mediated GTP hydrolysis required to inactivate Rab5-mediated protein trafficking functions^17^. A G protein binding site was proposed within the human p85α BH domain^29^ that was used as a guide to identify the residues required for Rab5 binding and regulation. Subsequent studies on numerous G protein–GAP complexes confirmed that many of the residues are within the Rab5–BH domain interface^28^. Since there is no crystal structure of the p85α BH domain – Rab5 complex, we modeled this interaction after the known crystal structure of the Cdc42GAP – Cdc42 complex^30^ by aligning the structure of the human p85α BH domain with that of Cdc42GAP and the structure of Rab5 with Cdc42 (Fig. 6a) as was done previously^10^. This model positions all the p85α BH domain residues shown to impact Rab5 binding (L191, V263) or regulation (R151, R274) on one surface of the domain in close proximity to Rab5 near the bound GTP analogue (Fig. 6b), consistent with them having a key role in regulating this interaction. In particular, R151 of the p85α BH domain superimposes with the arginine finger of Cdc42GAP and thus is positioned to act similarly.

Several bovine p85α BH domain mutations had little or no impact on Rab5 binding (R151D, K187A, L267D and L271D), suggesting that these residues are not required to mediate p85α BH domain–Rab5 contacts. Our previous mutational analysis of the p85α BH domain characterized the impact of R151A and R274A mutations on GAP activity and binding towards Rab5^17^. Both p85α BH mutations reduced the GAP activity towards Rab5, with the R274A mutation showing a substantially larger loss of GAP activity. Based on structural studies, R151 is positioned to function as the catalytic arginine finger for the p85α BH domain towards G proteins^30^ and the R151A mutant retains its ability to bind to both Rab5-GDP and Rab5-GTP^17^. Thus, the larger reduction in Rab5 GAP activity for the p85α-R274A mutation is likely due in large part to the corresponding loss of binding to Rab5-GTP (but retained binding to Rab5-GDP), also observed for the R274A mutant^17^.

We also mutated two residues within this proposed binding site that are conserved in the p85α BH domain from various species (e.g. human and bovine) and yet are highly divergent from other GAP domains (Supplementary Fig. S2)^30^. The divergent residues are L191 (R in other GAP domains) and V263 (N in other GAP domains) (Supplementary Fig. S2). Mutation of each of these p85α BH domain residues reduced the Rab5 binding and also the Rab5 GAP activity of p85α. Since L191 and V263 are highly conserved between p85α BH domains, and yet are very different than the polar residues Arg and Asn present at the corresponding sites within other GAP domains, this may explain why p85α is uniquely able to bind and regulate Rab5. Further, mutation of these residues had little or no impact on PTEN binding, suggesting that a distinct surface of the p85α BH domain mediates PTEN interactions.

The location of cancer patient-derived p85α BH mutations with significant impacts on Rab5 binding (I177, E217) and/or regulation (E137, I177, E217, E297) are shown in the modeled p85α BH domain: Rab5 complex (Fig. 6b). These mutation sites are located in regions of the p85α BH domain, well away from the Rab5 binding pocket. Further, crystal structure determinations for several of these mutant BH domains indicated that no major structural alterations were present. No significant differences in protein flexibility were observed for any of the mutant proteins (Supplementary Fig. S3), suggesting that the mutations did not alter the rigidity of the domain. Since the p85α BH domain mutants were analyzed for their impacts on Rab5 binding regulation in the context of the full-length p85α protein, it is possible that the p85α BH domain mutations influence the orientation or positioning of this domain relative to its other domains and in this way influences Rab5 binding and/or regulation.

The cancer-derived mutations within the SH3 domain of p85α (L30F, F69L, I82F) resulted in some reductions in Rab5 binding, with little impact on Rab5 regulation. Further, the L30F p85α mutation also affected PTEN binding. Given that the relative positions of the SH3 and BH domains within the ternary structure of the full-length p85α is not known, we can only speculate that the SH3 domain mutations may influence the interdomain interactions to impact the distinct PTEN and Rab5 binding sites within the p85α BH domain.

In summary, we have defined key residues within the p85α BH domain that bind and/or regulate the Rab5 GTPase. Rab5 plays a key role in membrane tethering during receptor-endocytosis and trafficking^14,45^. Defects in Rab5 regulation can be oncogenic due to alterations in cell signaling pathways including that of PI3K^33^ and can contribute to changes in cell adhesion, migration, invasion and metastasis^26,27^. Some of the patient-derived mutations within either the N-terminal half of p85α (i.e. SH3 or BH domains) were able to alter Rab5 and/or PTEN regulation suggesting that they may contribute to oncogenesis and tumor progression, in addition to the more common p85α mutations within the C-terminal half that deregulate p110α-PI3K activity. Patients with N-terminal p85α mutations may require different therapies than those with C-terminal mutations as a result of their impact on Rab5 and/or PTEN instead of p110α-PI3K.

## Methods

### Plasmids and mutagenesis

The glutathione S-transferase (GST)-Rab5 mutants (S34N and Q79L) have been described^46^. The GST- p85α plasmid, encoding bovine residues 1–724, has been described^47^. The GST-p85α BH domain fragment was generated by PCR amplification of the GST-p85α cDNA and subcloned into pGEX6P1. After Prescission protease cleavage from GST, the p85α BH domain (residues 105–319) was 24 kDa. The GST- p85α plasmid contains full-length human p85α (residues 1–724), fused in-frame after GST. Site-directed mutagenesis of p85α was carried out using the QuikChange method (Stratagene), according to the manufacturer’s directions. DNA sequencing to ensure that no additional mutations had been introduced verified the entire p85α coding region. Generation of the GST-PTEN plasmid has been described^11^.

### Pull-down binding assays

Pull-down assays with immobilized GST or GST-PTEN were allowed to bind purified precleared p85α protein (after cleavage from GST)^11^. Briefly, purified p85α protein (250 μg in 1 mL 20 mM Tris pH 8, 100 mM NaCl) was precleared by incubating with GST (200 μg) immobilized on glutathione Sepharose beads overnight at 4 °C to remove non-specific binding proteins. Each pull-down experiment used GST or GST-PTEN (5 μg each) immobilized on glutathione Sepharose bead incubated with precleared p85α protein (5 μg) in 1% milk in a total volume of 500 μl PBS (137 mM NaCl, 2.7 mM KCl, 4.3 mM sodium phosphate, 1.4 mM potassium phosphate, pH 7.3) for 30 minutes at room temperature. Beads were washed five times in 1 mL wash buffer (50 mM Tris, pH7.5, 150 mM NaCl, 1% NP-40). Each sample was resolved by SDS-PAGE and immunoblotted for p85α. Blots were quantified using the LI-COR Odyssey infrared imager, and the Odyssey v3.0 software was used to calculate the integrated intensity for quantification. Mutant binding values were normalized to that for wild type p85α binding on each blot by dividing the integrated intensity for the mutant by that for wild type p85α. Pull-down assays with immobilized GST or GST-Rab5 were carried out similarly after pre-loading the Rab5 with the indicated nucleotide according to the method detailed previously^48^. Bound p85α was detected after resolving samples by SDS-PAGE and transferring protein onto nitrocellulose, by immunoblotting with anti-p85α antibody (1:200; EMD catalogue number 05 217). Secondary antibodies linked to Infrared-dyes (IRDye800CW, IRDye680; LI-COR Biosciences) were used for detection and quantification using Odyssey software (v3.0). The results for all blots shown are representative of the results obtained from three independent experiments unless otherwise stated.

### Circular dichroism measurements

Circular dichroism spectra were recorded for the p85α BH domain mutants that showed reduced binding to PTEN as compared to the wild type p85α protein to ensure that protein folding was retained. Briefly, circular dichroism spectra from 200 nm to 280 nm (spectral bandwidth 1 nm, step size 0.5 nm) were obtained for p85α protein solutions (0.035–0.1 mg/ml) in 50 mM Tris pH 8, 150 mM NaCl, 100 μM TCEP (Tris(2-carboxyethyl) phosphine), 0.05% tween-20 using an Applied Photophysics Chirascan Plus CD Spectrometer at room temperature. At least four repeat scans were obtained for each sample and its buffer baseline. The averaged baseline spectrum was subtracted from the averaged sample spectrum and the net spectrum smoothed with Chirascan software.

### Rab5 GAP assays

GAP assays were used to determine the enhanced rate of GTP-hydrolysis mediated by Rab5-GTP upon addition of 8 μM p85α protein^17^. Rab5 (200 nM) was loaded with [α-^32^P]-GTP (0.89 pmol) in the presence of Mg^2+^ (10 mM) in GAP assay buffer (50 mM Tris pH 8.0, 150 mM NaCl, 2 mM EDTA, 1 mM DTT). Rab5 was allowed to hydrolyze the bound nucleotide to [α-^32^P]-GDP for 20 min at room temperature either alone (no p85α) or in the presence of wild type or mutant p85α. A stop solution containing 1% SDS, 25 mM EDTA, 25 mM GDP, 25 mM GTP was added at a ratio of 1:6 to the reaction, which was then incubated at 65 °C for 2 minutes. Nucleotides were resolved by thin layer chromatography on PEI Cellulose F plates and quantified by the Typhoon FLA 7000 phosphorimager with ImageQuant TL software v8.1.0.0 (GE Healthcare). Each experiment was repeated three times. The amount of hydrolysis of GTP by Rab5 alone was used as a control and was subtracted from the experimental results. A range of concentrations of p85α was tested – 2 μM, 4 μM, 8 μM, 16 μM, and 32 μM – and the 8 μM experiment was used to compare p85α mutant GAP activity to wild type. The Rab5 GAP activity of wild type p85α was typically 0.23 ± 0.02 mmol GTP hydrolyzed/min/mol of p85α, similar to what has been reported previously^17^. The data is reported as relative GAP activity to wild type p85α.

### PTEN lipid phosphatase activity assay

A phosphate release assay was used to determine the effects of wild type and mutant p85α on PTEN activity. Assays were performed by incubating His_6_-PTEN (1 μM) with Di-C8-PI3,4,5P_3_ lipid (200 μM; Echelon Biosciences) in the presence of wild type or mutant p85α (7.5 μM) in a final volume of 10 μl in phosphate release buffer (100 mM Tris-HCl pH 8.0, 1 mM DTT). All incubations were performed at 37 °C for 20 min and reactions were stopped with the addition of 100 mM N-ethylmaleimide (15 μl; Sigma). For detection, 20 μl of the reaction was combined with 80 μl BIOMOL Green (Biorad) in a 96-well plate and the color was allowed to develop for 20 min at room temperature. The absorbance was measured at 620 nm with a microplate reader. Each data point was assayed in duplicate, and all experiments were repeated five times. Values for buffer controls were subtracted from those for experimental samples and reported relative to PTEN activity in the presence of wild type p85α.

### Protein purification for crystallization

*E. coli* (BL21 DE3) expression of the bovine GST- p85α BH domain (105–319) wild type and mutants were induced with isopropyl ß-D-1-thiogalactopyranoside (IPTG; 0.1 mM) overnight at 25 °C^17^. Cell were pelleted and resuspended in lysis buffer (50 mM Tris pH 7.0, 150 mM NaCl, 1 mM EDTA, 1 mM DTT, 10 µg/mL aprotinin, 10 µg/mL leupeptin, and 1 mM 4-(2-aminoethyl)benzenesulfonyl fluoride hydrochloride (AEBSF)) with 1 µg/mL lysozyme, incubated at 4 °C for 1 hour. Sample viscosity was reduced via sonication on ice. Cell debris was pelleted by centrifugation and the supernatant was filtered through 0.8 µm Nalgene syringe filters (Thermo Scientific). GST-p85α protein fragments in the supernatant were incubated with glutathione Sepharose high performance media (GE Healthcare, column bed height 8.0 cm, column bed diameter 1.6 cm, column volume 16 mL) using an ÄKTA Purifier system. The sample was injected onto the column in phosphate buffered saline (PBS; 137 mM NaCl, 2.7 mM KCl, 4.3 mM Na_2_HPO_4_, 1.4 mM KH_2_PO_4_, pH 7.3), using a flow rate of 1 mL/min, pressure limit of 0.5 MP. The column was washed until the absorbance at 280 nm was less than 20 milli absorbance units. GST-p85α proteins were eluted from the column using 50 mM Tris pH 8.0, 150 mM NaCl, and 10 mM reduced glutathione. Fractions containing protein were pooled and dialyzed for 16 hours in Spectra/Por molecular porous membrane tubing (Spectrum Medical Industries Inc.; Los Angeles, CA; MW cut-off of 6000–8000 Da) against Prescission protease buffer (50 mM Tris pH 8.0, 150 mM NaCl, 1 mM EDTA). The p85α fragments were cleaved from GST by the addition of PreScission protease (GE Healthcare #27-0843-01) for 72 hours at 4 °C, according to the manufacturer’s instructions. GST was removed from the samples by passing the sample through the HR 16/10 glutathione Sepharose column as before, collecting the desired p85α protein fragments in the flow through.

The p85α samples were buffer exchanged using Millipore Amicon 10 kDa centrifugal filter units into buffer A (50 mM Tris pH 8.0, 50 mM NaCl, 2 mM DTT) and loaded onto a Source Q column (GE Healthcare, column volume 20.1 mL). Bound protein was eluted using buffer B (50 mM Tris pH 8.0, 1 M NaCl, 2 mM DTT) across 3 column volumes while collecting 1 mL fractions. Fractions were analyzed using SDS-PAGE analysis and Coomassie Blue staining to verify protein purity and yield. Fractions containing purified protein were pooled, concentrated and buffer exchanged into the desired storage buffer using Millipore Amicon 10 kDa centrifugal filter units. Proteins were stored at 4 °C.

### p85α BH domain protein crystallization and structure determination

Identification of initial crystal conditions was performed via preparation of hanging drop vapor diffusion screens of commercially available sparse matrix screening kits using 15 mg/mL p85α BH domain. These sparse matrix screens were set up using a GRYPHON robot (Art Robbins Instruments) which generated 0.4 µL drops by mixing 0.2 µL protein solution in crystal holding buffer (20 mM Bis-Tris Propane pH 6.5, 100 mM NaCl, 2 mM tris [2-carboxyethyl] phosphine hydrochloride [TCEP-HCl]) with 0.2 µL crystallization mother liquor in 96-well 2-well intelliplates (Art Robbins Instruments) with 100 µL of crystallization mother liquor deposited in the well reservoir. Conditions that yielded crystals for the p85α BH fragment and mutants were 0.1 M Sodium Cacodylate pH 6.0–6.2, 1.3–1.7 M Li_2_SO_4_, and 4–8% Glycerol.

X-ray diffraction data were collected at the Canadian Light Source (CLS, Saskatoon, SK) using the Canadian Macromolecular Crystallography Facility 08B1-1 beamline (bending magnet)^49^, or the Canadian Macromolecular Crystallography Facility 08ID beamline (small gap undulator)^50^. Cryoprotectant solution (0.1 M Sodium Cacodylate pH 6.0, 1.5 M Li_2_SO_4_, 18% [w/v] glycerol) was added to the sample crystal drops prior to crystal harvesting and data collection.

X-ray diffraction data (180°) were collected with a detector distance of 280 mm, in 0.5° or 1° wedges. X-ray detectors equipped at the beamlines were a Rayonix MX300 HE CCD for the CMCF-BM and a Rayonix MX300 CCD for the CMCF-ID. Processing of collected data was performed using HKL2000 software^51^ (Supplementary Table S1).

### Structure refinement using PHENIX and Coot software, and PyMOL image generation

Structure determination was performed using *PHENIX* software^52^. As the human p85α BH domain crystal structure has previously been solved^29^ we used molecular replacement with the human p85α BH domain (1PBW) as the initial model. Two molecules of the bovine p85α BH domain were located using *PHASER*. Starting atomic coordinates (with appropriate amino acid substitutions) were iteratively rebuilt using *Coot* and refined with *PHENIX* and *Refine*. Repeated cycles of phenix.refine and manual adjustments in *Coot*, including placement of solvent, were performed to further improve the structure. After refinement of the bovine wild type p85α BH structure, this was used as the starting structure for refinement of the p85α BH point mutant structures. Amino acids 113–297 of p85α were visible forming a homodimer within the crystal lattice. Residues 169–171 and 277–279 did not have visible electron density in either chain, and Pro 276 did not have visible electron density in Chain A. The root-mean-square difference on backbone atoms (RMSD) was determined for the overlay of the backbone atoms of the bovine and human p85α BH domain A chain monomers using Lsqkab in the CCP4 suite of programs. There was extra electron density at residue C146, consistent with the presence of either a sulfenic acid or S-nitrosocysteine residue. Hence the model contains sulfenic acid at this position. All B-plots were calculated using the CCP4 program “Baverage” using the final structures for the p85 BH Wild-Type, E137K, E217K, R262T, or E297K as input data.

Figures were generated using the PyMOL Molecular Graphics System (Version 1.4.1 Schrödinger, LLC).

### Statistical analyses

Assay results are expressed as means ± standard errors from at least 3 independent experiments, unless otherwise indicated. One-way analysis of variance (ANOVA) with post hoc Bonferroni tests were used for multiple comparisons with differences considered as statistically significant if *P* < 0.05.

### Availability of data and materials

Atomic coordinates and structure factors have been deposited at the RCSB depository (http://www.rcsb.org/pdb/explore.do): bovine p85α BH domain: wild type PDB ID# 6D81 and mutants E137K (PDB ID# 6D82), E217K (PDB ID# 6D85), R262T (PDB ID# 6D87) and E297K (PDB ID# 6D86). The materials generated and/or protocols used during the current study are available from the corresponding author on reasonable request.

## Electronic supplementary material

Supplementary Information
