# Supplementary material for: Patient-derived mutations within the N-terminal domains of p85α impact PTEN or Rab5 binding and regulation
Source: Sci Rep. 2018 May 8;8:7108. doi: 10.1038/s41598-018-25487-5 (PMC5940657; doi:10.1038/s41598-018-25487-5)
Supplement: Supplementary file 1 — Supplementary Information [file 41598_2018_25487_MOESM1_ESM.pdf]

Supplementary Information

Patient-derived mutations within the N-terminal domains of p85α impact PTEN or Rab5 binding and regulation

Paul Mellor[1], Jeremy D. S. Marshall[1,2], Xuan Ruan[1], Dielle E. Whitecross[1], Rebecca L. Ross[3], Margaret A. Knowles[3], Stanley A. Moore[2] and Deborah H. Anderson[1,2,4*]

[1]Cancer Research Group, University of Saskatchewan, 107 Wiggins Road, Saskatoon, Saskatchewan, S7N 5E5, Canada

[2]Department of Biochemistry, University of Saskatchewan, 107 Wiggins Road, Saskatoon, Saskatchewan, S7N 5E5, Canada

[3]Section of Experimental Oncology, Leeds Institute of Cancer and Pathology, St James's University Hospital, Leeds, United Kingdom

[4]Cancer Research, Saskatchewan Cancer Agency, 107 Wiggins Road, Saskatoon, Saskatchewan, S7N 5E5, Canada

Supplementary figure legends

Figure S1. Characterization of p85α mutant secondary structure.

A-C) Circular dichroism spectra comparing purified mutant p85α proteins to the control p85α wild type protein to ensure secondary structure is retained.

Figure S2. Sequence alignment of a several breakpoint cluster region (BCR)-homology (BH) domains encoding GTPase activating protein (GAP) domains. The BH domains of p85α human (H; accession number P27986; residues 113-301), bovine (B; accession number P23727; residues 113-301) and mouse (M; accession number P26450; residues 113-301) encoding GAP activity towards Rab5, compared to the corresponding human GAP domains that regulate other GTPases including BCR domain (accession number P11274; residues 1154-1248), Cdc42GAP (accession number Q07960; residues 242-431) and RacGAP1 (accession number Q9H0H5; residues 349-539). Residues involved in catalytic activity (R151, orange), Rab5 binding (red), or proposed Rab5 binding site (grey) are indicated. Patient-derived p85α BH domain mutations from endometrial cancers (black) and bladder cancers (brown) are also shown.

Figure S3. The flexibility of the mutant p85α BH domains is similar to that of the wild type protein. The B-factor values for each main chain atom of the BH domain structures from the wild type and mutant proteins were graphed and overlaid for comparison. Wild type (navy), E137K (grey), E217K (black), R262T (teal), E297K (green).

Figure S4. Full-length images of the cropped blots presented in the main figures.

Full-length images of Figure 1b.

Figure S5. Full-length images of the cropped blots presented in the main figures.

Full-length images of Figure 2a.

Figure S6. Full-length images of the cropped blots presented in the main figures.

Full-length images of Figure 3b and 3d.

Supplementary Table S1. Data collection and refinement statistics for p85α BH domain wild type and patient-derived mutants

	**p85α-BH wild type**	**p85α-BH E137K**	**p85α-BH E217K**	**p85α-BH R262T**	**p85α-BH E297K**
**Beamline**	CMCF-ID	CMCF-BM	CMCF-BM	CMCF-BM	CMCMF-BM
**Resolution range (Å)[b]**	50.0-2.25 (2.29-2.250)	50.0 - 2.40 (2.44 – 2.40)	50.0 – 2.20 (2.24 – 2.20)	50.0 – 2.70 (2.75 – 2.70)	50.0 – 2.30 (2.34 – 2.30)
**Space group**	$P2_12_12_1$	$P2_12_12_1$	$P2_12_12_1$	$P2_12_12_1$	$P2_12_12_1$
**Unit cell (a, b, c, α, β, γ)**	85.825, 91.737, 93.778, 90, 90, 90	85.364, 91.924, 93.274, 90, 90, 90	85.474, 91.647, 93.556, 90, 90, 90	85.499, 91.637, 93.574, 90, 90, 90	85.643 91.26 93.324, 90, 90, 90
**Total reflections**	264033	214849	280322	153261	244247
**Unique reflections[b]**	35631 (1747)	28798 (1419)	37700 (1851)	20681 (1024)	33073 (1627)
**Multiplicity[b]**	7.4 (7.2)	7.5 (7.6)	7.4 (7.5)	7.4 (7.5)	7.4 (7.5)
**Completeness[b] (%)**	99.2 (98.6)	99.1 (99.0)	99.7 (99.1)	99.3 (100.0)	100.0 (100.0)
**R-merge[a,b]**	0.094 (0.638)	0.070 (0.453)	0.060 (0.420)	0.089 (0.501)	0.069 (0.426)
**Mean I/sigma(I)**	24.2 (3.0)	31.7 (4.8)	36.9 (4.6)	23.3 (4.1)	31.4 (4.5)
**Refinement**					
**Resolution range (Å)[b]**	46.90-2.248 (2.309-2.248)	35.76-2.407 (2.493-2.407)	35.79 – 2.203 (2.263 – 2.203)	35.80 – 2.700 (2.842 – 2.700)	35.80 – 2.30 (2.369 – 2.301)
**Reflections [b]**	35,588 (2519)	28759 (2577)	37651 (2658)	20643 (2751)	33106 (2511)
**R-work[b,c]**	0.1957 (0.2268)	0.1971 (0.2309)	0.1988 (0.2275)	0.1952 (0.2287)	0.2064 (0.2432)
**R-free[b,d]**	0.2203 (0.2515)	0.2345 (0.2681)	0.2249 (0.2592)	0.2337 (0.2848)	0.2261 (0.2637)
**Number of non-hydrogen atoms**	2926	2938	2955	2895	2978
**macromolecules**	2840	2848	2850	2811	2888
**Number solvent atoms**	86	90	105	84	90
**Protein residues**	358	358	358	355	358
**RMS (bonds)**	0.004	0.005	0.005	0.003	0.003
**RMS (angles)**	0.79	0.82	0.80	0.73	0.75
**Ramachandran favoured (%)**	96.79	96.49	97.08	97.33	96.51
**Ramachandran outliers (%)**	0.87	1.17	0.58	0.00	4.39
**Rotamer outliers (%)**	1.55	2.50	0.93	4.08	1.88
**Wilson B-factor**	33.63	35.79	34.85	40.94	34.84

[a] $R_{merge} = \Sigma_{hkl} \Sigma_i \mid < I(hkl)_{obs} > - I(hkl)_{obs, i} \mid / \Sigma_{hkl, i} I(hkl)_{obs, i}$ where $I(hkl)_{obs, i}$ is the individual measurement of an hkl intensity and $< I(hkl)_{obs} > = \Sigma_i I(hkl)_{obs,i} /N$; where i = 1 to N individual reflections are measured.

[b] Values in parentheses correspond to the highest resolution shell.

[c] $R_{work} = \Sigma_{hkl} \mid |F_{obs}(hkl)| \mid - |F_{calc}(hkl)| \mid / \Sigma_{hkl} |F_{obs}(hkl)|$, where $|F_{obs}(hkl)|$ and $|F_{calc}(hkl)|$ are the observed and calculated amplitudes, respectively, for the structure factor **F(hkl)**.

[d] R_{free} is the equivalent of R_{work} for 5% of the reflections (randomly selected) which were not used in structure refinement.

a

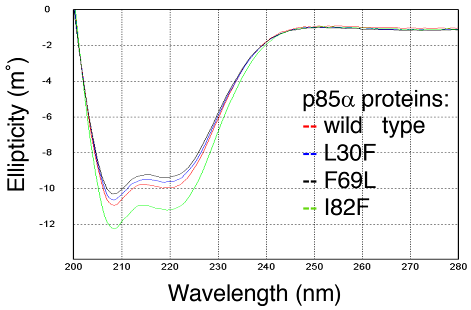
0
-2
-4
-6
-8
-10
-12
200
210
220
230
240
250
260
270
280
Ellipticity (m°)
Wavelength (nm)
p85α proteins:
wild type
L30F
F69L
I82F

b

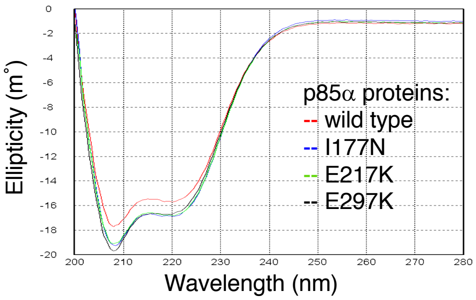
0
-2
-4
-6
-8
-10
-12
-14
-16
-18
-20
200
210
220
230
240
250
260
270
280
Ellipticity (m°)
Wavelength (nm)
p85α proteins:
wild type
I177N
E217K
E297K

c

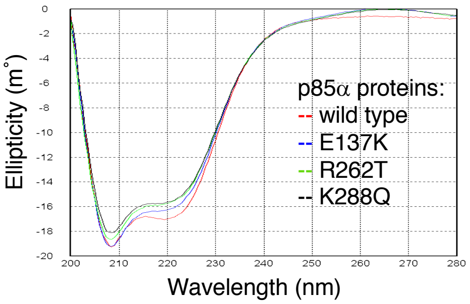
0
-2
-4
-6
-8
-10
-12
-14
-16
-18
-20
200
210
220
230
240
250
260
270
280
Ellipticity (m°)
Wavelength (nm)
p85α proteins:
wild type
E137K
R262T
K288Q

E137K R151

Sequence	1–40
p85alpha (H)	L T L P D L A E Q F A P P D I A P P L L I K L V E A I E K K G L E C S T L Y R T
p85alpha (B)	S T L P D L A E Q F A P P D V A P P L L I K L V E A I E K K G L E C S T L Y R T
p85alpha (M)	L P L P D L A E Q F A P P D V A P P L L I K L L E A I E K K G L E C S T L Y R T
BCR (H)	V K I A V V T K R E R S - - K V P Y I V R Q C V E E I E R R G M E E V G I Y R V
CDC42GAP (H)	V S L Q H L Q E K N P E Q E P I P I V L R E T V A Y L Q A H A L T T E G I F R R
RacGAP1 (H)	G M L A D F V S Q T S P - - M I P S I V V H C V N E I E Q R G L T E T G L Y R I

I177N K187

Sequence	41–80
p85alpha (H)	Q - S S S N L A E L R Q L L D C D T P S V D L E M - - I D V H V L A D A F K R Y
p85alpha (B)	Q - S S S N P A E L R Q L L D C D T A S L D L E M - - F D V H V L A D A F K R Y
p85alpha (M)	Q - S S S N P A E L R Q L L D C D A A S V D L E M - - I D V H V L A D A F K R Y
BCR (H)	S G V A T D I Q A L K A A F D V N N K D V S V M M S E M D V N A I A G T L K L Y
CDC42GAP (H)	S A N T Q V V R E V Q Q K Y N M G L P V D F D Q Y N - - E L H L P A V I L K T F
RacGAP1 (H)	S G C D R T V K E L K E K F L R - V K T V P L L S K V D D I H A I C S L L K D F

L191 E217K

Sequence	81–120
p85alpha (H)	L L D L P N P V I P A A V Y S E M I S L A P E V Q S S E E Y I Q L L K K L I R S
p85alpha (B)	L L D L P N P V I P V A V S S E L I S L A P E V Q S S E E Y I Q L L K K L I R S
p85alpha (M)	L A D L P N P V I P V A V Y N E M M S L A Q E L Q S P E D C I Q L L K K L I R L
BCR (H)	F R E L P E P L F T D E F Y P N F A E G I A L S D P V A K E S C M L N L L L S -
CDC42GAP (H)	L R E L P E P L L T F D L Y P H V V G F L N I D E - - - - S Q R V P A T L Q V L
RacGAP1 (H)	L R N L K E P L L T F R L N R A F M E A A E I T D - - - E D N S I A A M Y Q A V

Δ237-242 R262T V263 I267

Sequence	121–160
p85alpha (H)	P S I P H Q Y W L T L Q Y L L K H F F K L S Q T S S K N L L N A R V L S E I F S
p85alpha (B)	P S I P H Q Y W L T L Q Y L L K H F F K L S Q T S S K N L L N A R V L S E L F S
p85alpha (M)	P N I P H Q C W L T L Q Y L L K H F F K L S Q A S S K N L L N A R V L S E I F S
BCR (H)	- - L P E A N L L T F L F L L D H L K R V A E K E A V N K M S L H N L A T V F G
CDC42GAP (H)	Q T L P E E N Y Q V L R F L T A F L V Q I S A H S D Q N K M T N T N L A V V F G
RacGAP1 (H)	G E L P Q A N R D T L A F L M I H L Q R V A Q S - P H T K M D V A N L A K V F G

P270 M271 R274 K288Q E297K

Sequence	161–200
p85alpha (H)	P M L F R F S A A S S D N T E N L I K V I E I L I S T E W N E R - - - - - - - -
p85alpha (B)	P L L F R F P A A S S E N T E H L I K I I E I L I S T E W N E R - - - - - - - -
p85alpha (M)	P V L F R F P A A S S D N T E H L I K A I E I L I S T E W N E R - - - - - - - -
BCR (H)	P T L L R P S E K E S K L P A N P S Q P I T M T D S W S L E V M S Q V Q V L L Y
CDC42GAP (H)	P N L L - - W A K D A A I T L K A I N P I N T F T K F L L D H Q G E L F - - - -
RacGAP1 (H)	P T I V A H A V P N P D - P V T M L Q D I K R Q P K V V E R L L S L P L E Y W -

Supplemental Figure S3 - Mellor

a Chain A

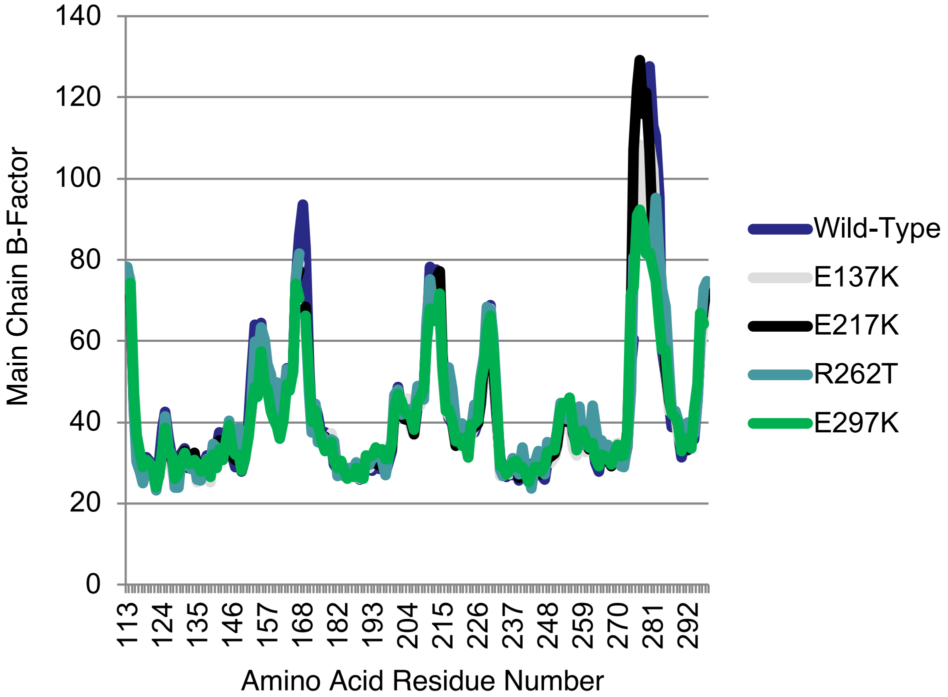

b Chain B

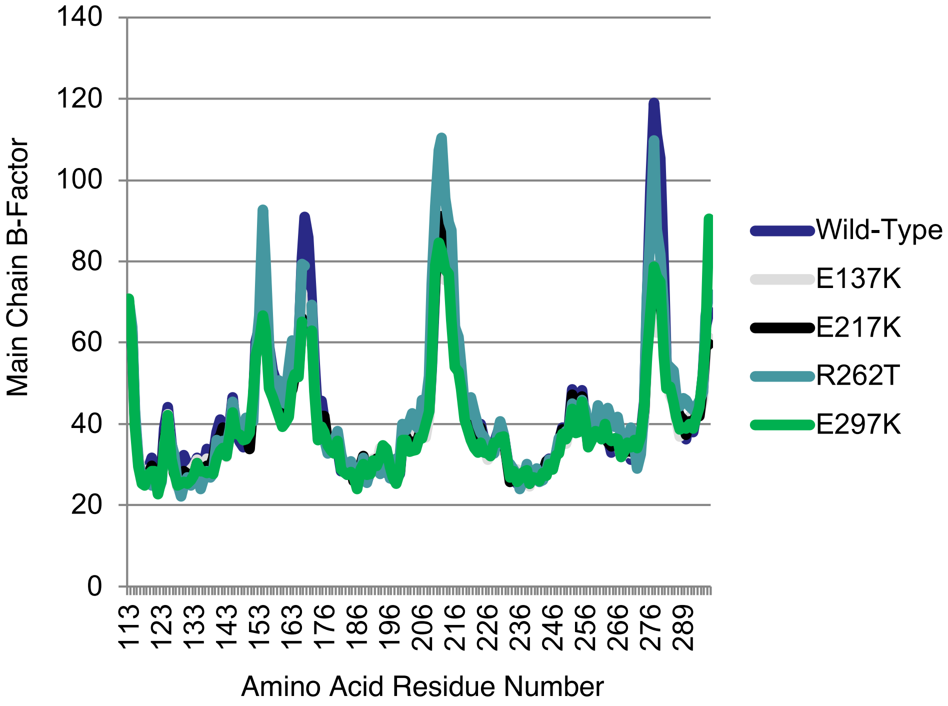

Supplemental Figure S4 - Mellor

Full-sized: Figure 1a

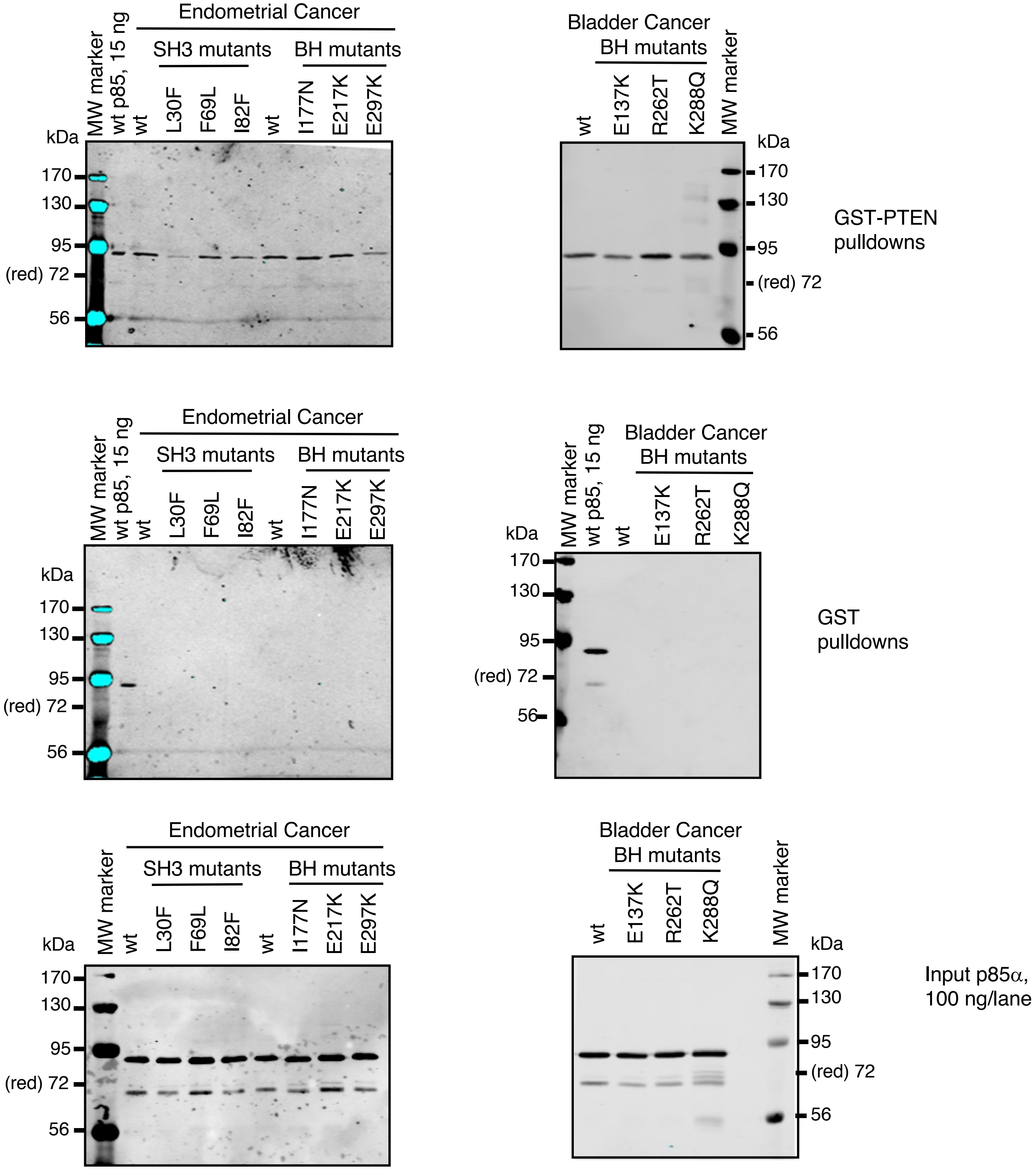

all anti-p85α blots

Full-sized: Figure 2a

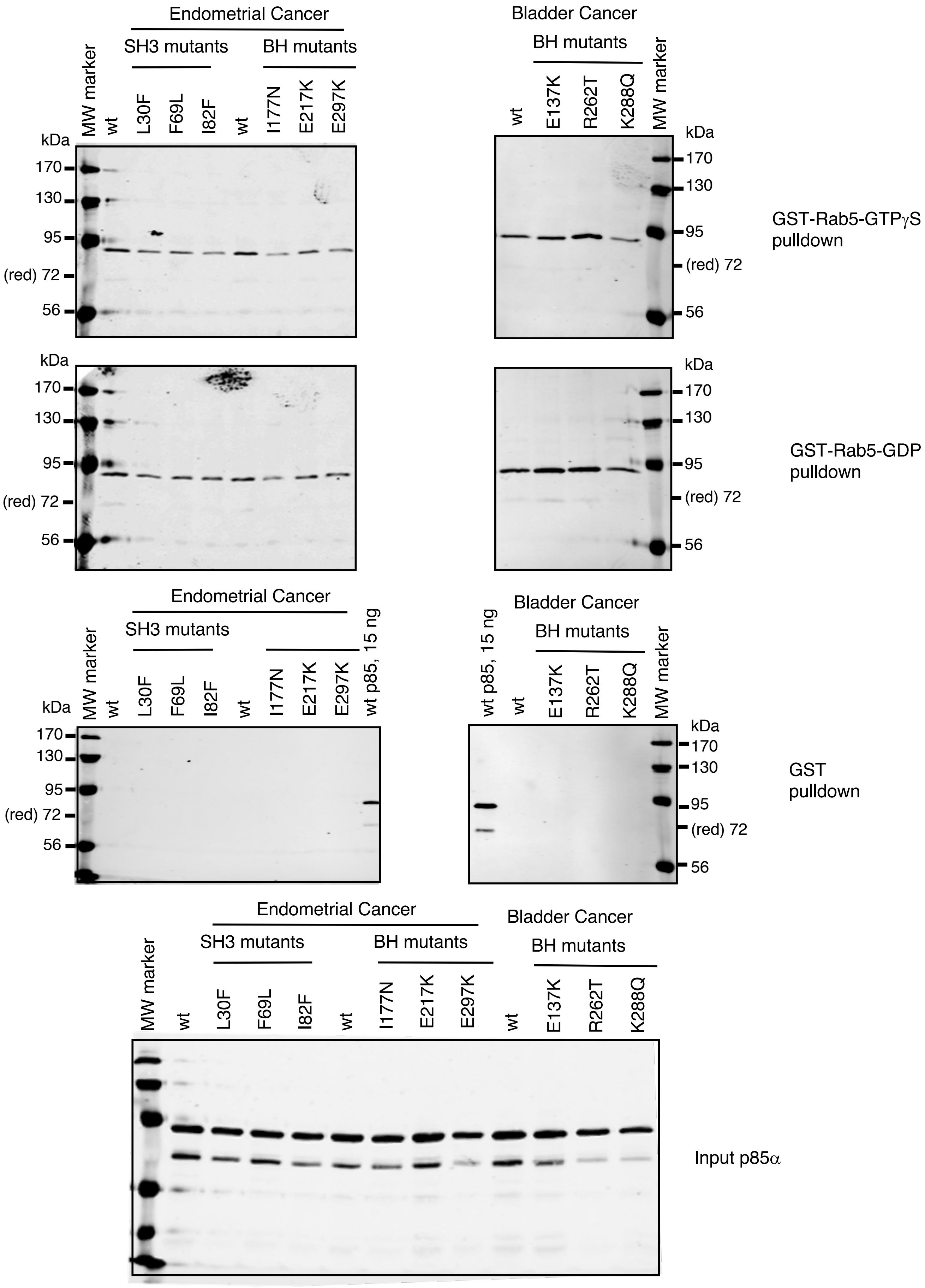

all anti-p85α blots

Full-sized: Figure 4b

a

GST-Rab5 pull-downs with purified p85α proteins

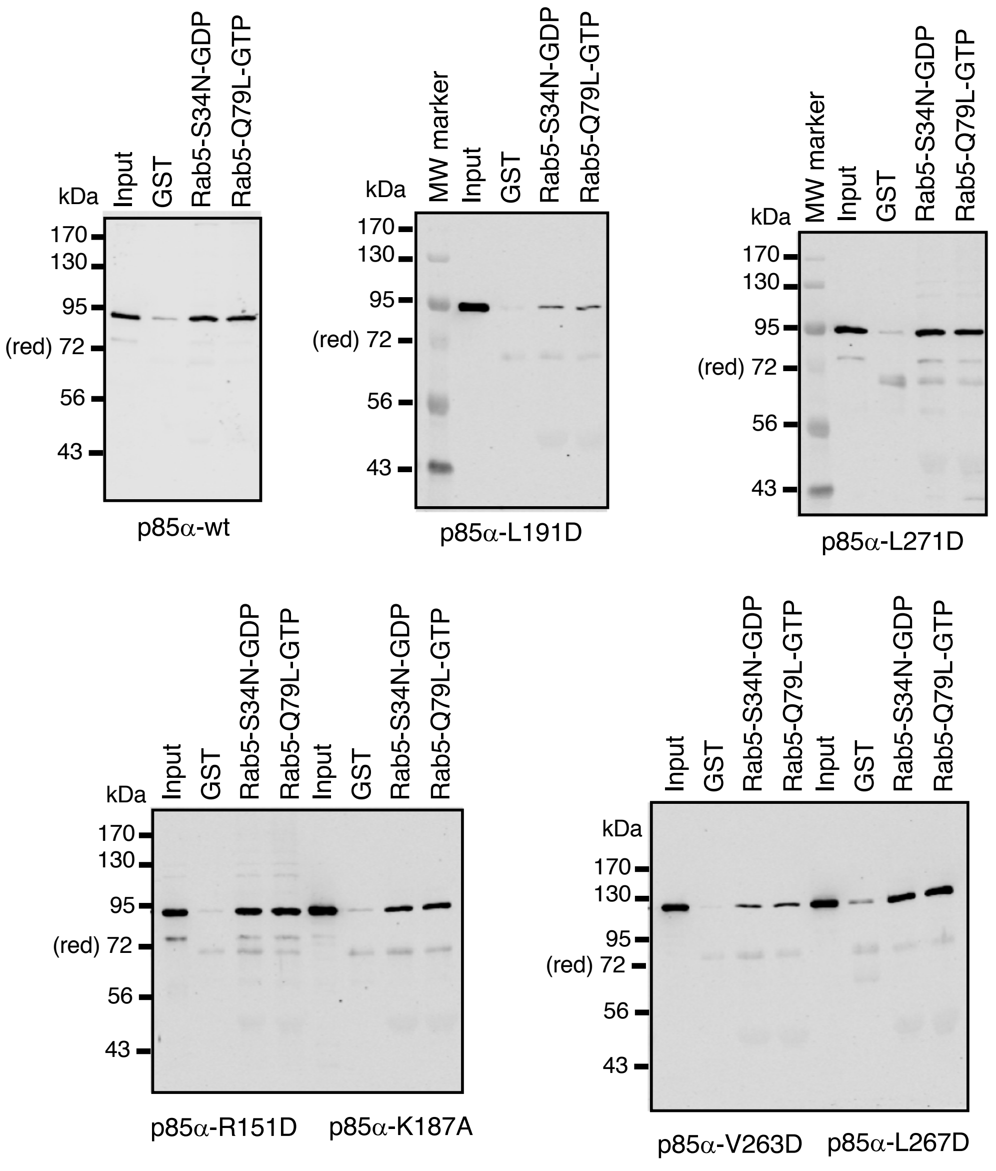

b

Full-sized: Figure 4d

GST-PTEN pull-downs with purified p85α (Rab5 binding site mutants)

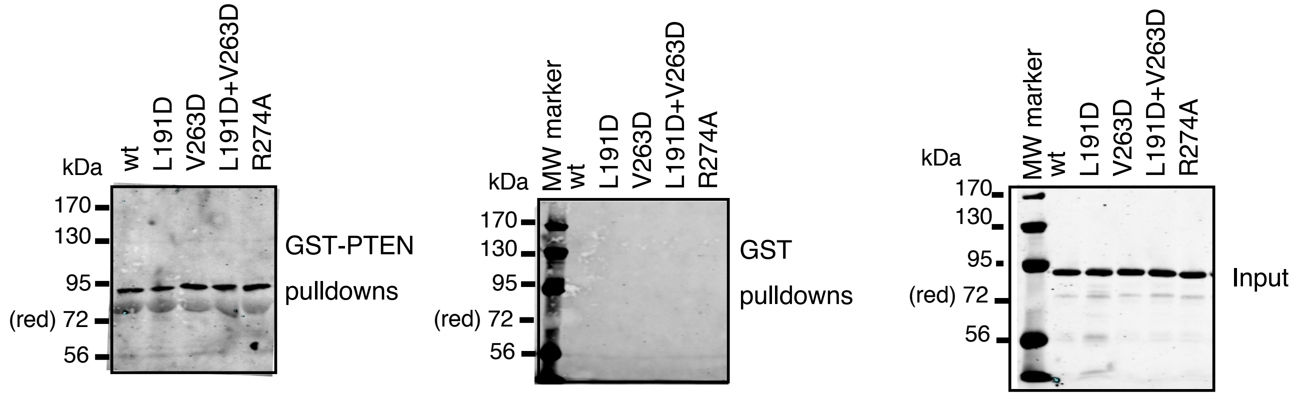

all anti-p85α blots
